# Quantitative genome-wide association analyses of receptive language in the Danish High Risk and Resilience Study

**DOI:** 10.1186/s12868-020-00581-5

**Published:** 2020-07-07

**Authors:** Ron Nudel, Camilla A. J. Christiani, Jessica Ohland, Md Jamal Uddin, Nicoline Hemager, Ditte Ellersgaard, Katrine S. Spang, Birgitte K. Burton, Aja N. Greve, Ditte L. Gantriis, Jonas Bybjerg-Grauholm, Jens Richardt M. Jepsen, Anne A. E. Thorup, Ole Mors, Thomas Werge, Merete Nordentoft

**Affiliations:** 1 Institute of Biological Psychiatry, Mental Health Centre Sct. Hans, Mental Health Centre Sct. Hans, Mental Health Services Copenhagen, Roskilde, Denmark; 2 iPSYCH, The Lundbeck Foundation Initiative for Integrative Psychiatric Research, Copenhagen, Denmark; 3 Mental Health Centre Copenhagen, University of Copenhagen Hospital, Copenhagen, Denmark; 4 Section for Biostatistics, Department of Public Health, University of Copenhagen, Copenhagen, Denmark; 5 Mental Health Centre for Child and Adolescent Psychiatry–Research unit, Mental Health Services in the Capital Region of Denmark, Copenhagen, Denmark; 6 Psychosis Research Unit, Aarhus University Hospital–Psychiatry, Aarhus, Denmark; 7 Center for Neonatal Screening, Department for Congenital Disorders, Statens Serum Institut, Copenhagen, Denmark; 8 Center for Neuropsychiatric Schizophrenia Research and Center for Clinical Intervention and Neuropsychiatric Schizophrenia Research, Mental Health Services in the Capital Region of Denmark, Copenhagen, Denmark; 9 Department of Clinical Medicine, Faculty of Health and Medical Sciences, University of Copenhagen, Copenhagen, Denmark

**Keywords:** Linguistics, Genetics, GWAS, Parent-of-origin, Receptive language, Danish High Risk and Resilience Study

## Abstract

**Background:**

One of the most basic human traits is language. Linguistic ability, and disability, have been shown to have a strong genetic component in family and twin studies, but molecular genetic studies of language phenotypes are scarce, relative to studies of other cognitive traits and neurodevelopmental phenotypes. Moreover, most genetic studies examining such phenotypes do not incorporate parent-of-origin effects, which could account for some of the heritability of the investigated trait. We performed a genome-wide association study of receptive language, examining both child genetic effects and parent-of-origin effects.

**Results:**

Using a family-based cohort with 400 children with receptive language scores, we found a genome-wide significant paternal parent-of-origin effect with a SNP, rs11787922, on chromosome 9q21.31, whereby the T allele reduced the mean receptive language score by ~ 23, constituting a reduction of more than 1.5 times the population SD (P = 1.04 × 10^−8^). We further confirmed that this association was not driven by broader neurodevelopmental diagnoses in the child or a family history of psychiatric diagnoses by incorporating covariates for the above and repeating the analysis.

**Conclusions:**

Our study reports a genome-wide significant association for receptive language skills; to our knowledge, this is the first documented genome-wide significant association for this phenotype. Furthermore, our study illustrates the importance of considering parent-of-origin effects in association studies, particularly in the case of cognitive or neurodevelopmental traits, in which parental genetic data are not always incorporated.

## Background

Language is one of the most fundamental human traits, and, as such, has been studied from many different angles, in disciplines such as philosophy, linguistics, psychology, anthropology, neuroscience and, more recently, genetics.

In the previous century, many studies showed that linguistic traits were heritable, i.e. that the genetic variation across individuals can explain some of the phenotypic (exhibited trait, in this case, linguistic ability) variation in the studied population. The investigated traits in those studies were either manifestations of atypical language development or of specific language skills in the general population [1], although the genetic factors influencing the two do not have to be the same. These insights were obtained through various study designs, such as twin studies and pedigree studies, which did not examine molecular genetic data.

With advances in molecular genetic techniques and the establishment of samples with available language-related phenotypes, studies were performed whose goal was to identify genes (or genetic variants) associated with linguistic ability. It was a series of studies of one extended pedigree which ultimately reported the first gene implicated in a speech and language disorder, *FOXP2* [2–4]. Despite the fact that the disorder involving *FOXP2* was monogenic (caused by a disruption of a single gene), the precise mechanism through which this gene caused the speech and language disorder is still unknown, and many subsequent studies examined the gene’s molecular function, its molecular evolution and its involvement in vocal communication in other species [5]. The disorder present in the aforementioned extended pedigree was rare and monogenic; non-synonymous variants in *FOXP2* were not the cause of a common form of language impairment, namely specific language impairment (SLI) [6]. SLI is diagnosed when language development is deficient in the absence of known neurological or (other) neurodevelopmental abnormities [7]. This includes, for example, a discrepancy between verbal and non-verbal intelligence. However, in recent years, the diagnostic criteria have been changed, and more focus is given to the clinical picture of the child. As a consequence, a new label was adopted: developmental language disorder [8]. A discrepancy between verbal and non-verbal intelligence is not required for the latter diagnosis.

The first genome-wide screen for susceptibility loci for SLI was a non-parametric linkage analysis performed by the SLI Consortium [9], and it was later extended by the addition of more families to the cohort [10]. These studies identified a region on chromosome 16 linked to nonword repetition skills (reflecting phonological short-term memory) and a region on chromosome 19 linked to expressive language skills, in children with SLI. While a receptive language score was one of the quantitative phenotypes examined in the SLI Consortium linkage screen, no chromosomal regions were found to be significantly linked to it. However, in a later multivariate linkage analysis, receptive language traits did contribute to linkage signals e.g. on chromosomes 19 and 10 [11]. Another linkage study identified a region associated with reading impairment in children with SLI on chromosome 13 [12]; however, unlike the SLI Consortium studies, this study used parametric linkage analysis in large pedigrees. The same locus was investigated further in a following study [13]. Using an isolated population with a high rate of language impairment, linkage analyses under several models highlighted a region on chromosome 7 [14]. Regions on chromosomes 10 and 13 were identified in a more recent linkage study [15].

With the advent of dense marker maps and single-nucleotide polymorphism (SNP) arrays towards the end of the 2000s, and in relation to theoretical considerations in complex disease studies, disease-gene mapping strategies in the field of human genetics shifted from linkage studies towards genome-wide association studies (GWAS) [16]. In 2013, two GWAS were published which reported suggestive SNP associations with language-related traits in the general population [17, 18]. In 2014, the first GWAS of SLI reported a significant association exhibiting paternal parent-of-origin effects in a locus on chromosome 14 [19]. The same locus was later associated with reading-related traits, also displaying parent-of-origin effects in two dyslexia cohorts, albeit with discordant trends between the phenotypes [20]. A different approach, also employed in a GWAS design, was to test for association with principal components generated from both language and reading traits [21]. A receptive language phenotype was also investigated in a previous GWAS, although parent-of-origin effects were not modeled [22]. Expressive vocabulary in toddlers was also included in a GWAS design, which found a genome-wide significant association [23]. Complementary to genome-wide studies, other studies examined specific regions and/or variants following prior evidence of relevance to language traits. This included targeted association analyses of linkage regions [24], an exome sequencing study of the aforementioned isolated population [25] and an immunogenetic study of SLI [26], the latter of which also examined parent-of-origin effects.

A parent-of-origin effect denotes a change in the way an allele may influence a trait dependent on which parent it was inherited from. There are good reasons to examine parent-of-origin effects in general, and in studies of language-related traits and disorders in particular: firstly, they may account for some of the so-called “missing heritability” (the difference between disease heritability estimates and the variance explained by significant GWAS associations across published studies) [27]. A recent study illustrated that these effects can be missed in traditional designs, and that, in some cases, the same variant may have opposing effects on a given trait when inherited paternally or maternally [28]. A later study extended this observation to several quantitative traits [29]. Of note, this effect has also been observed in an association between a specific *HLA*-*B* allele and receptive language in SLI [26]. Secondly, parent-of-origin effects have been reported for several neurodevelopmental and psychiatric conditions [30]. Importantly, the latter include SLI [19, 26], as previously mentioned, and Williams syndrome [31], both of which involve abnormal development singling out language; while SLI involves impaired language development with other domains relatively unaffected, Williams syndrome is typically presented as involving a cognitive impairment which does not include linguistic ability (people with Williams syndrome are, in fact, hypersocial and hyperverbal), or, in other words, as a “mirror image” of SLI [32], although this view is, perhaps, too simplistic or inaccurate [33–35]. A recent family-based GWAS of autism spectrum disorder (ASD) highlighted 18 parent-of-origin effects, but the model employed in that study additionally included child genetic effects and maternal effects, all modeled simultaneously [36].

At this point it would be useful to discuss the expressive language-receptive language divide, as several of the aforementioned studies examined or found significant results for only one type of language trait. Expressive language refers to language production, and receptive language refers to language comprehension. An illustration of what can be measured by scores of expressive or receptive language ability can be found in the Clinical Evaluation of Language Fundamentals [37], which was used by the SLI Consortium in their studies. Expressive language subtests include, for example, formulating sentences (the child needs to formulate a sentence based on visual stimuli) and recalling sentences (the child needs to imitate a sentence produced first by the examiner), while receptive language subtests include, for example, semantic relationships (the child needs to answer questions correctly based on a spoken sentence) and oral directions (the child needs to point to pictured objects in response to directions). While it is easy to outline the different domains of expressive and receptive language, the distinction is not necessarily straightforward when considering real observations. For example, expressive and receptive language scores were found to be highly correlated in the SLI Consortium cohort [9]. Another issue in this regard is with the classification of children into groups based on the type of problems they display. For example, children “moved” from a cluster of expressive language disorder to expressive-receptive language disorder on follow-up only one year later [38]. Nonetheless, many standardized language tests maintain this distinction and provide separate scores for these domains. Leonard discusses this distinction in the context of SLI in more detail [39].

The above studies provided insight into the genetic basis of language, but genome-wide studies of language-related traits and language disorders remain scarce, relative to studies of other cognitive phenotypes and neurodevelopmental disorders. Studies in which parent-of-origin effects are modeled are even scarcer, as parental genotype data are not always available. In this study, we attempted to fill in some of these gaps by examining receptive language (which has not been the focus in follow-up studies to the first genome-wide screen of SLI) in a relatively large family cohort, and by examining parent-of-origin effects as well as child genetic effects in a GWAS design.

## Results

**Fig. 1:**
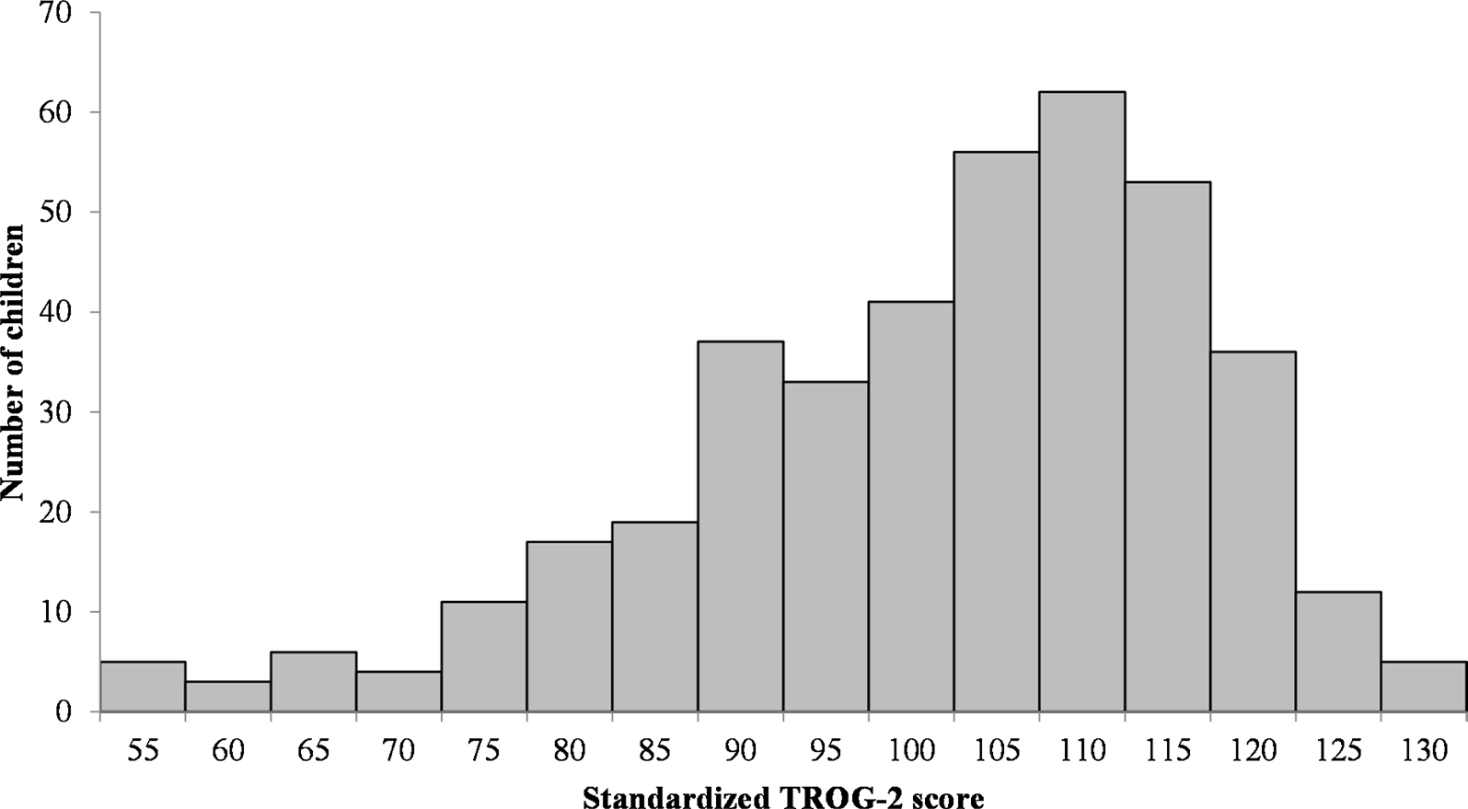
The distribution of the standardized TROG-2 scores across all children in the study

In total, 400 children had Test for Reception of Grammar 2 (TROG-2) scores in our dataset. The distribution of the TROG-2 scores is shown in Fig. 1. The mean score across all 400 children was 101.2 with a standard deviation (SD) of 15.6, close to the population values/norms of 100 and 15, respectively. The distribution of these scores was not completely Gaussian. The means (SDs) within the ASD, attention deficit/hyperactivity disorder (ADHD) and index family subgroups were lower at 94.9 (± 16.8), 92.7 (± 17.3) and 99.8 (± 16.5, based on 243 children, as one child did not have a TROG-2 score), respectively. It should be mentioned that, in some cases, children who received an ASD diagnosis might have received it in part due to having low language ability, as mentioned in the Methods section; however, not all children with ASD had TROG-2 scores below the population mean for their age, and some indeed had scores above the population mean.

In the discovery stage, we found a genome-wide significant association in the paternal parent-of-origin test; the top hits in the other two tests showed only suggestive association: rs12297354 in the general test (P = 7 × 10^−6^), and rs9397338 in the maternal parent-of-origin test (P = 8 × 10^−6^). Manhattan and QQ plots for the discovery analyses are shown in Additional file 1: Figure S1. Associations that are at least suggestive (P ≤ 10^−5^) from all three tests can be found in Additional file 2: Table S1.

**Fig. 2:**
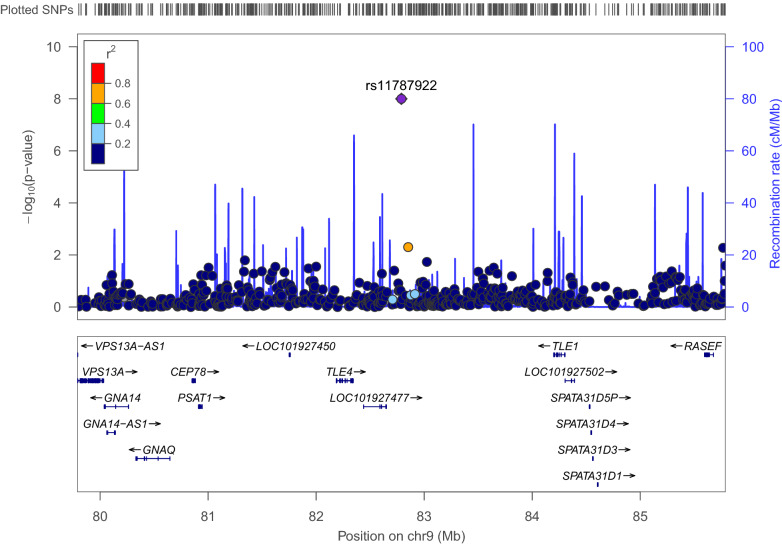
A regional association plot showing the top SNP in the paternal parent-of-origin analysis and surrounding SNPs and genes

The top hit in the paternal parent-of-origin test was with an intergenic SNP on chromosome 9:82,788,836, rs11787922 (Fig. 2). In our analysis, when examining alleles inherited from the father, the T allele (the minor allele), was associated with a reduction of 23.05 in the TROG-2 score (P = 1.04 × 10^−8^), which is more than 1.5 SD away from the population mean. The frequency of the T allele in our entire sample was 7.54%. It was 5.88%, 10.64% and 6.98% in the ASD, ADHD and index family children subgroups, respectively. The T allele was not genome-wide significantly associated in the general test (its effect was − 9.46, P = 9 × 10^−6^) or in the maternal parent-of-origin test (its effect was − 0.287, P = 0.927), but it showed suggestive association in the former.

To assess the evidence for a paternal association at the above locus further, we performed five post hoc tests. The first test assessed whether there was a significant difference between the maternal and paternal alleles; it is possible that, when looking only at paternally inherited alleles, the association is significant, and that, when looking at maternally inherited alleles, it is not (or vice versa). However, that alone does not mean that the difference itself between the associations with paternal and maternal alleles is significant. QTDT has a special test for that, and our first post hoc test confirmed that the difference was indeed significant in this case (P = 5 × 10^−6^). In our second post hoc test, we repeated the association test having added covariates for ASD, ADHD and for whether the family was an index family or a control family, as we had observed lower scores (on average) in children from all three groups compared to all children in the study. While the covariates explained some of the reduction in the TROG-2 score, the genetic effect was still large (effect of T allele on TROG-2 score = − 21.3, P = 3.25 × 10^−8^). When using a covariate for sex, the effect of T allele on TROG-2 score was − 22.9, (P = 1.13 × 10^−8^).

In the fourth post hoc test, where scores were transformed to approach normality and scaled, the association signal between rs11787922 and receptive language remained significant (P = 8.33 × 10^−7^), with the T allele being associated with a reduced score, but the effect was somewhat diminished at − 1.3 SD of the transformed score. Variance component models are sensitive to departure from normality; we used them in our model to account for relatedness in 11 families with a sibship of 2. If we remove one sibling from each of these families (keeping children with the T allele, if only one sibling had it, and otherwise randomly choosing one) and do not include variance components in the model, the results are similar to the original, with an effect of the T allele = − 22.9 (P = 2 × 10^−8^).

Lastly, we used an independent method of assessing parent-of-origin effects in unrelated individuals. Using this method, we observe an association signal for rs11787922 (P = 0.0016). This method relies on unrelated individuals and was designed with larger samples in mind, and we also lost power by excluding individuals. Nonetheless, even with this method, we observed some signal at this locus. Table 1 includes a summary of all post hoc tests.

**Table 1:** Results of the post hoc tests for rs11787922 (the original test is included as a point of reference)

Test	Description of the test	Test statistic	Effect of T allele on TROG-2 score, where relevant	*P* value
Original test from the paternal parent-of-origin discovery analysis	This test compares two models, where only the full model includes the effect of only paternally-derived alleles. The likelihoods of the two models are then compared using a likelihood ratio test (χ^2^ statistic).	χ^2^ = 32.76; df = 1	− 23.05	1.04 × 10^−8^
Test for the difference between paternal and maternal alleles	This test compares two models, where the null model already has the X = within = between family component from the “total” association model, and the full model includes, in addition to X, the effect of only maternally-derived alleles. The likelihoods of the two models are then compared using a likelihood ratio test (χ^2^ statistic).	χ^2^ = 20.7; df = 1		5 × 10^−6^
Paternal parent-of-origin test with covariates for ASD, ADHD, and index family status	This test compares two models, where only the full model includes the effect of only paternally-derived alleles. Both models include the effects of the covariates. The likelihoods of the two models are then compared using a likelihood ratio test (χ^2^ statistic).	χ^2^ = 30.55; df = 1	− 21.3	3.25 × 10^−8^
Paternal parent-of-origin test with a covariate for sex	This test compares two models, where only the full model includes the effect of only paternally-derived alleles. Both models include the effect of the covariate. The likelihoods of the two models are then compared using a likelihood ratio test (χ^2^ statistic).	χ^2^ = 32.61; df = 1	− 22.9	1.13 × 10^−8^
Paternal parent-of-origin test using Z-scores of the transformed TROG-2 scores	This test compares two models, where only the full model includes the effect of only paternally-derived alleles. The likelihoods of the two models are then compared using a likelihood ratio test (χ^2^ statistic).	χ^2^ = 24.28; df = 1		8.33 × 10^−7^
A test for the presence of a parent-of-origin effect in unrelated individuals	This test compares the trait variance of the heterozygous group to those of the homozygous groups.	$\frac{{\hat{b}}}{{SE_{b} }}\;\sim \;N\;\left( {0,1} \right) \; = \;0. 2 6 6 4/0.0 8 9 8$		0.0016

## Discussion

Our GWAS identified a genome-wide significant association for receptive language with a paternal parent-of-origin effect of a SNP on chromosome 9, rs11787922, supported by both a family-based association method and an independent method of assessing parent-of-origin effects which is based on the variance of the trait in unrelated individuals. This association was not driven by the presence of two other neurodevelopmental disorders in which language may be impaired, namely ASD and ADHD, nor was it driven by the parents’ having a diagnosis of schizophrenia or bipolar disorder, despite the fact that the receptive language scores across individuals with ASD, ADHD or whose parents had the above psychiatric diagnoses were lower on average compared to the entire cohort. This suggests that the association of this SNP with receptive language is not part of a larger behavioral phenotype encompassing ASD, ADHD, familial risk of schizophrenia or bipolar disorder. In this context, it would be useful to note that a study of polygenic risk which used the same cohort used in this study [40] found that a polygenic risk score (PRS) trained on a previous GWAS of SLI was not predictive of the risk of ASD or ADHD in this cohort, but it was predictive of the risk of SLI. The latter resulted in a Nagelkerke’s R^2^ (adjusted for prevalence and the proportion of cases in the sample) of > 5%, compared to values of close to 0% for ASD and ADHD (see footnote in the Methods section for more information about this). A different way of estimating additive genetic overlaps between disorders is estimating genetic correlations between them. This can be achieved in several ways e.g. by using family data or through examining genetic and phenotypic variation across unrelated individuals, the latter being more similar to the PRS analyses. Earlier studies, which examined genetic correlations across ASD and ADHD or specific traits associated with the two disorders, reported moderate to high significant genetic correlations [41, 42], but SNP-based methods did not obtain similar estimates: they were either lower [43] or non-significant [44].

To our knowledge, this is the first genome-wide significant association reported for receptive language; a previous GWAS of receptive language (which did not model parent-of-origin effects) found only suggestive associations [22].

Our top SNP has not been highlighted in past genetic studies of language or other cognitive traits. Therefore, we surveyed the literature and examined the region surrounding the SNP for possible involvement in relevant traits. Interestingly, the protein-coding gene that is closest to the SNP is *TLE4* (Fig. 2), which is involved in neural development [45, 46]. The gene is involved in cell determination and differentiation in neurogenesis, together with other genes of the same family, working in different combinations of expression patterns during neurogenesis. *TLE4*, specifically, is expressed in neural progenitor cells in the early embryonic stage.

Through PhenoScanner [47], we found a proxy SNP for rs11787922, namely rs10512097 (r^2^ = 0.85 in Europeans, not included in our GWAS), which was a suggestive expression quantitative trait locus (eQTL) for *IMP5* (also called *SPPL2C*) (P = 7.18 × 10^−6^) [48]. Deletions of this gene have been reported for some forms of intellectual disability and developmental delay [49, 50], although de novo loss-of-function variants in a nearby gene were enough to cause a similar phenotype [51]. Recently, *SPPL2C* has been highlighted in a G × E study which examined the influence of common variants on language through low-frequency hearing ability [52].

Our top SNP, rs11787922, lies in a region encompassed by several pathogenic copy-number variants (CNVs) (found with the ClinGen track on UCSC Genome Browser) associated with various forms of developmental delay, including a CNV identified in an individual with delayed speech and language development (ClinVar accession number: VCV000148901.1). This CNV, however, was quite large (chromosome 9:71,130,848-86,285,142) and contains many genes, and so it could, at most, provide suggestive evidence for the region around the SNP’s being associated with a language phenotype. A survey of the literature found that the chromosomal band encompassing the SNP on chromosome 9 (i.e. 9q21.31), as well as adjacent bands, were implicated in several studies of phenotypes such as developmental delay, language delay and intellectual disability. At times these were part of a broad syndrome, namely Gorlin syndrome, which typically involves 9q22 and the *PTCH1* gene [53], but there were cases of individuals with CNVs in that region, not encompassing *PTCH1,* that were still associated with language problems, including associations with receptive language [54]. A study of nine individuals of whom eight showed severe speech and language delay identified deletions on 9q21 (mean size = 7.14 Mbp), of which six involved 9q21.31 [55]. Among the genes deleted across all deletion cases in that study were *RORB*, *TRPM6*, *NMRK1* and *OSTF1*. Of note, *RORB* was reported to be involved in verbal intelligence [56].

While there seems to be some evidence for the involvement of the locus on chromosome 9 in language-related phenotypes, one further point to consider is that our top association displayed a paternal parent-origin effect. Information regarding imprinted regions in the human genome is not readily available in online databases (imprinting, or silencing of one parental allele, being one of the major mechanisms underlying parent-of-origin effects). A “Catalogue of Imprinted Genes and Parent-of-origin Effects” exists [57], but it is quite small and has not been updated since 2016. Another database, Geneimprint [58], also contains a small number of entries, and they are limited to specific genes only. However, it is sometimes possible to find information in cytogenetic studies which could provide some insight regarding imprinting of genomic regions. For example, for 9q22, some deletions (not all involving *PTCH1*) associated with language delay or intellectual disability are of paternal origin, suggesting that the paternal allele is expressed in that region [59, 60]. This does not mean that the maternal allele is *not* expressed. Further insight can be gained by considering uniparental disomy (UPD) cases, i.e. scenarios in which both chromosomes of the same kind (or chromosomal segments) in the child come from one parent. If a region is paternally or maternally imprinted, then a UPD involving it may result in an abnormal phenotype, either due to having too much gene expression from two non-imprinted chromosomes, or insufficient gene expression, if both chromosomes are imprinted. One study [61] reported the case of a child with neurodevelopmental problems who had a paternal UPD from 9q21.33 onwards (a triplication of 9q21.11–q21.33, a region which included our top SNP, was also observed). This UPD involved inheriting two copies of a segment of one of the homologous chromosome 9’s from the father (isodisomy), which means that the phenotype may potentially be caused by either homozygosity or imprinting. Thus, there is some indirect evidence suggesting that the implicated region on chromosome 9 may be imprinted. It should also be noted that loci may display parent-of-origin effects if they interact with imprinted regions or genes, even if they are not imprinted themselves [62], which implies that even if the top SNP in our analyses is not in an imprinted region, it might still display parent-of-origin effects through interaction with imprinted genes or loci elsewhere in the genome.

Some of the suggestive associations (Additional file 2: Table S1) are also worth mentioning. For example, rs4632856 (P = 4 × 10^−6^ in the paternal parent-of-origin test) was shown to be an eQTL for *PHIP* [63] (P = 2.36 × 10^−8^), a gene which has been implicated in intellectual disability and developmental delay [64, 65]; rs3731769 (P = 0.00001 in the maternal parent-of-origin test) was suggestive (P = 4.95 × 10^−6^) in a study of autism [66]. Interestingly, it exhibited a paternal parent-of-origin effect. This opposite parental effect is similar to the one observed across the SLI and dyslexia studies, as mentioned earlier.

Our study had several limitations. Firstly, the genotyping array used in this study included both common and rare variants, the latter having been identified through studies of psychiatric disorders or included from an exome chip. Unfortunately, due to these variants being rare, most of them were filtered out for having low minor allele frequencies during QC; nearly half of the genotyped variants were removed and thus not included in downstream analyses. This also means that the genome-wide coverage is lower than in studies which used genotyping arrays with more common variants. Also related to this is the fact that we used software not designed to work with imputed dosage data or extremely large marker datasets. Secondly, cohorts with data on receptive language and genotyped parents (both mothers and fathers, to be able to test for both parent-of-origin effects and the difference between them) are scarce. Specifically, even though samples meeting those criteria do exist, they are often ascertained for having language disorders or related neurodevelopmental conditions, meaning that the receptive language scores are already likely to be quite below the population mean, which could potentially mask an effect observed in non-ascertained samples, and/or they may also comprise fewer families, as is the case for some of the previously mentioned studies. Our association thus requires replication in a suitable, independent sample. Lastly, the effect size of the top SNP in our study is quite large (the T allele was associated with a reduction of 23.05 in the TROG-2 score). In the general test of association (without parent-of-origin effects), the effect of the T allele was a reduction of 9.46 in the TROG-2 score, and it was not genome-wide significant. While we could not find relevant examples of studies employing parent-of-origin tests, the result in the general test is of the same order of magnitude (i.e. 1 < X < 10 for standardized scores with a mean of 100 and a SD of 15 in some studies, or 0.5 SD < X < 1 SD in other studies) as observed in associations between *CNTNAP2* variants and language traits in children with SLI [67], intelligence in a general population twin cohort [68], and handedness in individuals with dyslexia [69]. The fact that this effect appears smaller and less significant in the general test, assuming a true parent-of-origin effect is present, is in line with the results of the aforementioned studies which investigated parent-of-origin effects across several quantitative traits. However, we cannot rule out an overestimation of this effect or the winner’s curse [70, 71].

## Conclusions

We report a genome-wide significant association with receptive language in a sample not selected for language impairment. The association displays a parent-of-origin effect, whereby the minor allele reduces the language test score by more than 1.5 times the population SD, when inherited from the father. Our results contribute to the scientific literature and shed light on a relatively understudied research topic–the genetic underpinnings of linguistic ability. Moreover, our study illustrates the importance of considering parent-of-origin effects in genetic studies, which are often not modeled, in part due to constraints in data availability. In the same vein, we hope that our study will encourage further investigations into this and similar phenotypes.

## Methods

### Participants

The cohort used in this study comes from the Danish High Risk and Resilience Study–the VIA 7 study [72]. The VIA 7 study recruited children around age 7 and their biological parents. Families were selected on account of having one or two parents with a diagnosis of either schizophrenia spectrum psychosis or bipolar disorder (hereafter index families) or as control families, in which neither parent had schizophrenia or bipolar disorder (hereafter control families). *NB*: in other VIA 7 publications “index” may refer to the biological parents themselves who were selected for having a psychiatric diagnosis as well as to matched control parents (the non-index parent being the other biological parent). However, here, “index” refers to the families of parents who have a psychiatric diagnosis, and “control” refers to families of parents who do not have a psychiatric diagnosis. The families in the study were recruited from across Denmark. Just under half of the families lived in densely populated areas, with no significant differences in urbanization between index families (neither schizophrenia nor bipolar disorder) and control families. The parents in the schizophrenia group were younger and had lower levels of education compared to the other two groups. Both biological parents in the index families had lower levels of functioning and were less often in employment or enrolled in a study program [73]. In the genetic analyses, we removed individuals of non-European ancestry and their relatives, as explained below. The children were administered numerous tests and interviews, focusing on cognitive, behavioral, social and psychomotor measures and assessed for psychopathology.

A subset of the families in VIA 7 provided DNA samples, which were subsequently genotyped on the Illumina PsychChip v1.1. This subset was used in this study. Following genetic quality control (QC), individuals from 429 families remained, consisting of child-parents trios, child-parent duos, or only children, and, in a minority of families, a sibling as well (also, occasionally children might have been removed due to QC or not genotyped, in which case the parents might have been kept in the pedigree if they themselves passed QC, e.g. for more accurate allele frequency calculations). Due to the nature of the analyses, the precise number of children used in the association test with a given marker may differ across markers, as explained later in this section, but we note the precise number of informative children in the test for the top association observed and for the suggestive associations in Additional file 2: Table S1.

### Language data and other phenotypic measures

The test used in this study was the Test for Reception of Grammar (TROG-2) [74]. This test measures receptive language skills by presenting children with blocks of four sets of pictures. In each set of pictures, only one picture fully corresponds to a sentence uttered by the examiner. The child must choose the correct picture in every set to have “passed” the block correctly. Scores comprising the number of correct blocks were age-standardized according to the norms from the Danish manual. Children also underwent screening with the Danish version of the Schedule for Affective Disorders and Schizophrenia for School-Age Children (K-SADS) [75]. Children who received a probable or a definite diagnosis for ASD or ADHD based on the K-SADS were defined as cases for their respective diagnosis/diagnoses (children who did not have a probable or a definite diagnosis were defined as controls for each diagnosis separately) [76]. All diagnostic assessments of children who were suspected of having ASD or ADHD were reviewed by a specialist in child and adolescent psychiatry, and data from other measures available in the VIA 7 study might have been taken into consideration when making the diagnosis, including data on language ability. We used these indications to construct covariates in a post hoc test as described later. Out of the entire sample of children used in this study, 17 were diagnosed with ASD, 47 were diagnosed with ADHD and 244 came from an index family, as defined above.

### Genetic data and quality control

The QC steps for the genetic data are described in detail in a recent study of PRS for language impairment [40]. QC was performed with PLINK v1.9b5.2 [77]. The main differences between the dataset used in the PRS study and the one used in the present study are (i) that the present study did not exclude parents or siblings from the analyses, as the analyses were family-based, unlike the PRS analyses, (ii) that markers in the major histocompatibility region were not removed, and (iii) that duplicate markers (based on position) were removed (in the PRS analyses these would have been removed during the clumping procedure, if still present after earlier filtering steps and in the unlikely event that the same duplicate set of markers was included in both the base and target datasets). Due to the requirements of the software used in the present study, “dummy” individuals (i.e. individuals for whom genetic data were not available) were added to the pedigree so that all children had two parents in each nuclear family. Since by default PLINK checks for Mendelian errors only in trios, this resulted in a small number of additional errors found in some child-parent duos. No child-parent duos not otherwise removed during other QC steps showed an excess of errors large enough (> 1%) to have been removed for this alone, and only a small number of markers that were not removed previously were flagged for excess errors. While we confirmed that this issue did not have any major impact on the results of the PRS analyses in the previous study,^1^ we chose to remove the offending genotypes and markers from the present study, as it examined one marker at a time. Apart from the individuals removed for excess Mendelian errors (N = 10), individuals and markers were also removed following the other main steps, which are repeated here in brief form for easy reference: initial QC on raw genetic data: individuals with low call rates or discordant sex information were removed in the first step (N = 18), as were SNPs with a Gentrain score < 0.3. QC with PLINK: SNPs with > 5% missing data were removed (all remaining individuals had < 5% missing data). Individuals with extreme heterozygosity rates (with a threshold of ± 3 SD from the sample mean) were removed (N = 21). Genetic ancestry was estimated in a principal component analysis (PCA). The threshold for the exclusion of samples was 2 SD above or below the VIA 7 mean for either PC1 or PC2, using the VIA 7 samples and the CEU, CHB, JPT and YRI HAPMAP samples to create the PC space [78]. Individuals of divergent ancestry were removed along with their relatives (N = 36, the remaining individuals clustered near the European (CEU) reference individuals), as were individuals who exhibited cryptic relatedness (the Pi-hat threshold for the exclusion of individuals not known to be related was 0.185) or who were less related to family members than expected from pedigree information (N = 13). A Hardy–Weinberg Equilibrium *P* value threshold of 1 × 10^−6^ was employed for QC-passing SNPs, as well as a minor allele frequency threshold of 1%. In total, 299,604 autosomal SNPs passed all QC steps. Positions in the text and tables are in hg19.

### Discovery analyses

The software package QTDT v2.6.1 [79] was used for conducting the statistical genetic analyses (QTDT stands for quantitative transmission-disequilibrium test, although the tests employed here were not TDTs, as explained below). MERLIN v1.1.2 [80] was used for estimating identity by descent scores for each marker to be used by QTDT. Three types of analyses were performed: a total test of association using all family data (qtdt -at), a paternal parent-of-origin analysis (qtdt -at -op) and a maternal parent-of-origin analysis (qtdt -at -om). The “total association” model was used for all the above tests, as it is more powerful in the absence of population stratification (individuals of divergent ancestry had been removed from our dataset in the PCA step during QC). In this model, a combined between/within family component “X”, denoting the between/within effect on the means, is tested (X is the effect size reported for the QTDT analyses). In the null model this component is fixed to 0, and in the full model it is estimated from the data. The likelihoods of these two models are then compared resulting in a χ^2^ statistic (in most cases). We included variance components in all models (-wega), incorporating an environmental component, a polygenic component and an additive major locus component. This allowed for the use of families with multiple children. In the two parent-of-origin analyses, the same model was used, except that only paternally inherited alleles and maternally inherited alleles were used in the paternal parent-of-origin analysis and maternal parent-of-origin analysis, respectively. The phenotype used in the discovery analyses was the standardized TROG-2 score. LocusZoom [81] was used to plot the region surrounding the top association.

### The power of a QTDT analysis

The power of a QTDT analysis depends on factors such as: the marker allele frequencies, the effect size, the linkage disequilibrium between the marker and the quantitative trait locus, the number of genotypes in the analysis (determined by the number of children/siblings), whether parental genotypes are included or not, and the significance level. Power analyses for QTDT are carried out through simulation and were performed in several studies focusing on the method itself or on how it compares with other association methods. In the original QTDT paper, assuming a maximum D’, h^2^ of 0.1, risk allele frequency of 0.5, significance level of 0.001 and including parental genotypes, a sample of 480 children (families with a sibship of 1) resulted in a power estimate of 97.4% [79]. Another study reported a power of 74% with N = 200, h^2^ of 0.1, and a risk allele frequency of 0.3 [82]. However, it is important to keep in mind two main ways in which our analyses differ from these studied examples: (i) studies which looked into the power of a typical QTDT analysis employed the orthogonal model and not the total association model, the latter of which was used in our study, and (ii) they did not examine parent-of-origin models. These two factors are expected to suggest that our analyses should have greater power, all other things being equal, because: (i) the power to detect a parent-of-origin effect in trios tends be higher than the power to detect a child genetic effect, given equal sample sizes, if the model is correct [83], and (ii) in the absence of population stratification, the QTDT total association model has greater power than the orthogonal model [84].

### Post hoc tests

For the top association from the discovery analyses, we performed five additional tests: four tests were performed with QTDT as well: the first test checked whether the difference between the paternal and maternal alleles was significant (qtdt -at -ot).

The second test repeated the original analysis in which the top SNP was highlighted, incorporating covariates for a diagnosis of ASD, a diagnosis of ADHD, and whether the family was an index family or a control family. These were included as “dummy variables” having a value of 0 (control for ASD, ADHD/control family) or 1 (case for ASD, ADHD/index family). We also repeated the test with a covariate for sex (third test). This was done separately due to the sex bias observed in ASD and ADHD (to avoid correlation between the covariates).

The fourth test repeated the original analysis but used Z-scores of transformed TROG-2 standardized scores (Z-scores were used, because the transformed scores could have extreme values). The method for the transformation, the Yeo-Johnson transformation [85], and the parameter lambda = 2.762526, were selected with the bestNormalize package v1.4.3 [86] and employed with the VGAM package v1.1-2 [87] in R v3.6.3 [88]. This test was performed because QTDT assumes normality of the phenotype scores, and the distribution of the scores in our sample was not completely Gaussian. We therefore transformed the scores to approach normality. It is worth noting that the original QTDT method paper as well as several publications mentioning the assumption of normality and/or the effect of violating it discussed in this context the orthogonal model presented by Abecasis et al. in the QTDT paper [79, 89, 90]. As previously mentioned, the model we used was the total association model. In fact, the QTDT program provides a way to account for non-normality using a permutation procedure, but this is not implemented for the total association model in QTDT. Some differences between the two models should be noted: the total association model is not a TDT and makes use of more children (the difference in sample size between the orthogonal model and the total association model may be quite large, in favor of the latter). In particular, in addition to this model using information from more children, the total association model for parent-of-origin effects specifically is less constricted in terms of parental genotypes: the orthogonal parent-of-origin model uses children whose both parents are genotyped and where one parent is homozygous, or whose mother and father have different genotypes. In addition, when paternal parent-of-origin effects are tested, the father must be heterozygous and, when maternal effects are tested, the mother must be heterozygous. The total association parent-of-origin model uses, in addition to the above group of children, all children with at least one homozygous parent, even if the other parent has a missing genotype [91]. With regards to the top SNP in our analyses, the orthogonal model could use only 23 children (fewer than the default minimal number of informative children), whereas the total association model used 353 children, making it much more powered. This also illustrates the fact that individuals, and thus phenotype distributions, could vary greatly for each tested marker, especially when testing for parent-of-origin effects. Nonetheless, since the effect of a violation of normality in the total association model is not known, we decided to repeat the test following the transformation of the scores across all children. It should be mentioned that transforming trait values does not necessarily improve the way in which the model behaves and may limit the interpretation of the results [92–94], but we thought it best to include both estimates in the paper for the sake of completeness.

Additionally, we employed a newer approach for the detection of parent-of-origin effects for quantitative traits in unrelated individuals (fifth test). To this end we used the “POE method” as implemented in QUICKTEST v0.99b [95]. In this analysis, 391 unrelated children were used, the same sample as used in the PRS study [40] (TROG-2 scores were available for 389 children of those 391 unrelated children). The underlying principle of this approach is the following: assuming that a locus with alleles A and B has an additive effect on a phenotype (the two alleles have different effects on the quantitative trait), the three genotype groups AA, AB and BB will differ in their mean phenotype scores. If a parent-of-origin effect is present, then the heterozygous group (individuals with genotype AB) will consist of two subgroups, namely individuals with paternal A and maternal B and individuals with maternal A and paternal B, which should have different means, thereby increasing the variance of the heterozygous group as a whole. If there is no parent-of-origin effect at this locus, then the two heterozygous subgroups should not be distinguishable in this respect. This method provides a formal test for this by comparing the variance of the heterozygous group to those of the homozygous groups. It should be noted that his method does not indicate what type of parental effect (i.e. paternal or maternal) is present, because parental genotypes are not available to it; rather, it provides statistical evidence for the presence of such an effect.

## Supplementary information

**Additional file 1: Figure S1.** Manhattan and QQ plots for the discovery analyses.

**Additional file 2: Table S1.** Suggestive associations (P ≤ 0.00001) from the discovery analyses.

## Data Availability

Access to the dataset used in the current study are available from the corresponding authors upon reasonable request.

## Abbreviations

ADHD: Attention deficit/hyperactivity disorder
ASD: Autism spectrum disorder
CNV: Copy-number variant
eQTL: Expression quantitative trait locus
GWAS: Genome-wide association study
K-SADS: Schedule for Affective Disorders and Schizophrenia for School-Age Children
PCA: Principal component analysis
PRS: Polygenic risk score
QC: Quality control
QTDT: Quantitative transmission-disequilibrium test
SD: Standard deviation
SLI: Specific language impairment
SNP: Single-nucleotide polymorphism
TROG-2: Test for Reception of Grammar 2
UPD: Uniparental disomy

## Footnotes

1 For an example of this effect: the adjusted Nagelkerke’s R^2^ for the model for the SLI phenotype was 5.21% (with P < 0.05), for ASD it was 0.015%, and for ADHD it was 0.009%. The R^2^ for height was 0.019%; following the removal of some SNPs and Mendelian errors in duos, the values were 5.09% (with P < 0.05), 0.017%, 0.009% and 0.01%, respectively (using the dataset termed Correction 1 in the paper).
