# Supplementary material for: Quantitative genome-wide association analyses of receptive language in the Danish High Risk and Resilience Study
Source: BMC Neurosci. 2020 Jul 7;21:30. doi: 10.1186/s12868-020-00581-5 (PMC7341668; doi:10.1186/s12868-020-00581-5)
Supplement: Supplementary file 1 — Additional file 1: Figure S1. Manhattan and QQ plots for the discovery analyses. [file 12868_2020_581_MOESM1_ESM.pdf]

a

b

c

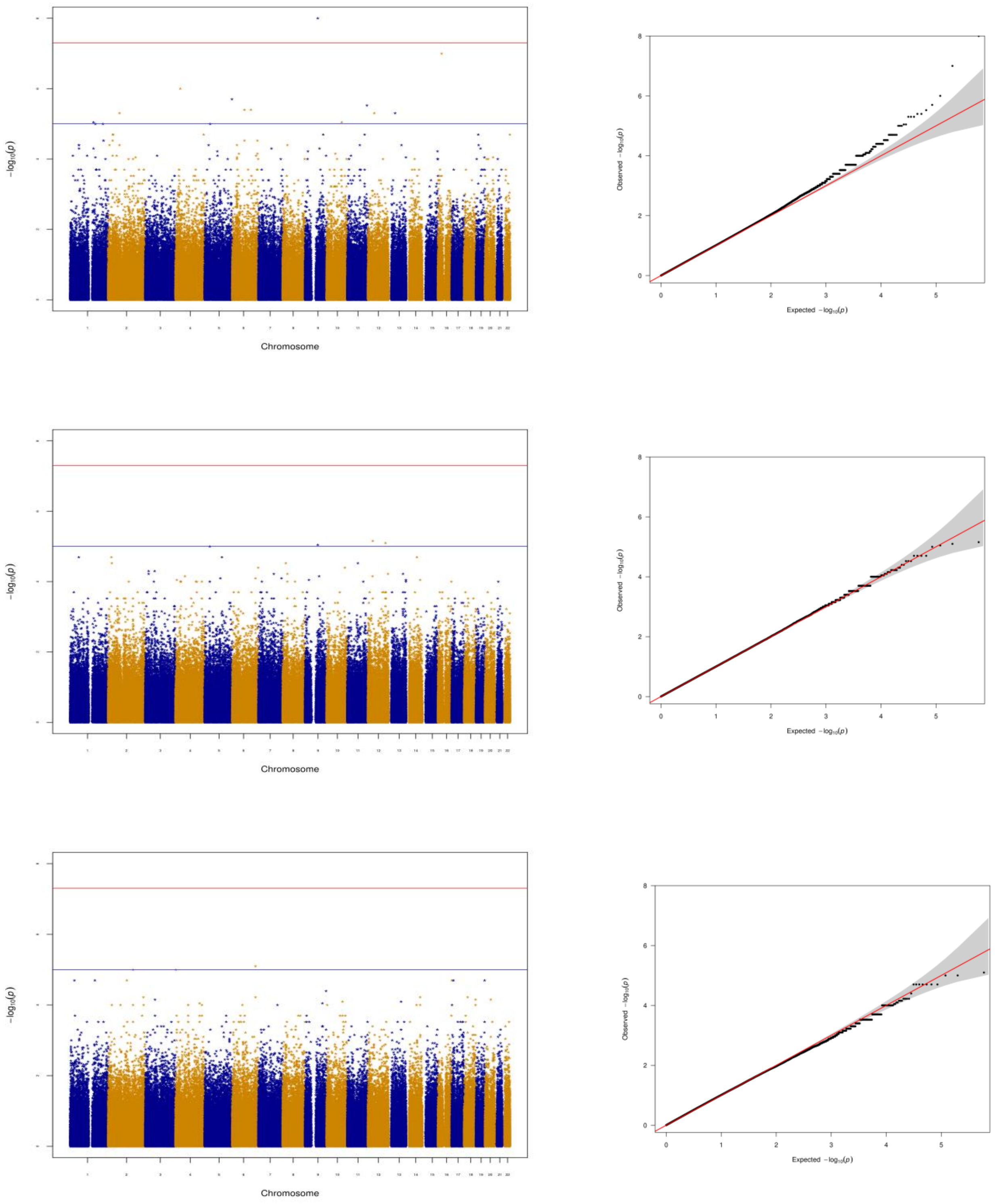

Figure S1: Manhattan and QQ plots for a) paternal parent-of-origin analysis; b) general test; c) maternal parent-of-origin analysis. In the Manhattan plots, the red line signifies genome-wide significance ($P=5\times10^{-8}$) and the blue line signifies the suggestive threshold ($P=10^{-5}$). The gray area in the QQ plots represents the 95% confidence interval.
